# Heterologous Expression of the *StCML50* Gene Enhances Drought Tolerance in Transgenic *Arabidopsis*

**DOI:** 10.3390/plants15030417

**Published:** 2026-01-29

**Authors:** Xinglong Su, Jia Wei, Junmei Cui, Xianglin Sun, Jinjuan Ma, Zhenzhen Bi, Yuhui Liu, Zhen Liu, Yongwei Zhao, Yajie Li, Feng Zhao, Jiangping Bai, Panfeng Yao, Chao Sun

**Affiliations:** 1 College of Agronomy/State Key Laboratory of Aridland Crop Science, Gansu Agricultural University, Lanzhou 730070, China; suxl@st.gsau.edu.cn (X.S.); weij@st.gsau.edu.cn (J.W.); cuijm@gsau.edu.cn (J.C.); 1073324120586@st.gsau.edu.cn (X.S.); 1073324120613@st.gsau.edu.cn (J.M.); bizz@gsau.edu.cn (Z.B.); lyhui@gsau.edu.cn (Y.L.); liuzhen@gsau.edu.cn (Z.L.); baijp@gsau.edu.cn (J.B.); 2 Seed Industry Research Institute of Gansu Provincial University, Gansu Agricultural University, Lanzhou 730070, China; 3 Dingxi Agricultural Science Research Institute, Dingxi 743000, China; zhaoyonwe1988@163.com (Y.Z.); 15095476815@163.com (Y.L.); 4 Institute of Economic Crop and Beer Material, Gansu Academy of Agricultural Science, Lanzhou 730070, China; zhaof@gsagr.cn

**Keywords:** potato, CMLs, drought stress, antioxidant enzymes

## Abstract

Calmodulin-like proteins (CMLs) are key mediators of plant calcium signaling and participate in abiotic stress responses, but their functions in potato remain poorly understood. Here, we systematically identified 62 *StCML* genes in potato via genome-wide analysis, which were phylogenetically clustered into seven clades and unevenly distributed across 12 chromosomes. Synteny analysis indicated that tandem and segmental duplications drove *StCML* family expansion, while promoter cis-element analysis suggested their involvement in phytohormone signaling and stress responses. Transcriptomic data showed *StCMLs* exhibited tissue-specific expression (high in roots, flowers, stamens) and were transcriptionally induced by drought, salt, and abscisic acid (ABA). Heterologous overexpression of *StCML50* in *Arabidopsis* enhanced drought tolerance, as evidenced by improved germination, root elongation, and survival compared to wild-type. Physiologically, *StCML50* overexpression increased proline accumulation, boosted antioxidant enzyme (SOD, CAT, POD) activities, and reduced malondialdehyde (MDA) levels under drought. Additionally, transgenic lines showed increased ABA sensitivity. This study provides insights into the potato *CML* gene family’s evolution and regulatory mechanisms, offering a valuable genetic resource for potato stress tolerance improvement.

## 1. Introduction

Plants are subjected to the synergistic effects of a variety of abiotic and biotic stresses during growth and development, including pathogen infestation, temperature extremes, and environmental stresses such as drought [1]. These stressors significantly reduce the yield, quality and agricultural productivity of plants by inhibiting their normal developmental processes [2]. In response to adversity stress, plants have evolved a complex regulatory network involving key physiological and biochemical processes such as phytohormone dynamic homeostasis, cellular signaling, and gene expression reprogramming [3]. Therefore, analyzing the functions of drought-resistant genes and their molecular regulatory mechanisms is of great theoretical value for breeding new high-yielding and drought-resistant crop varieties.

Calcium ions (Ca^2+^), as a key second messenger molecule, are involved in the transduction and amplification processes of multiple signaling pathways through dynamic changes in their cytoplasmic concentrations [4]. External stimuli can trigger a transient oscillatory signal of intracellular Ca^2+^concentration, which is recognized by specific Ca^2+^-binding proteins to initiate downstream response reactions [5]. Ca^2+^-binding proteins in plants are categorized into four main groups: calmodulin (CaM), calmodulin-like proteins (CML), calcium-dependent protein kinase (CDPK), and calmodulin phosphatase B-like proteins (CBL) [6,7,8]. Among them, CMLs, a plant-specific subclass of Ca^2+^-binding proteins, feature variable EF-hand domains (1–6) while lacking other functional motifs [9]. These Ca^2+^ sensors play crucial roles in plant signaling pathways. To date, genome-wide characterization of CML gene families has been reported in multiple species, including *Arabidopsis thaliana* [10], *Oryza sativa* [11], *Vitis vinifera* [12], *Brassica napus* [13], *Solanum lycopersicum* [14], *Medicago sativa* [15], *Ginkgo biloba* [16], *Chrysanthemum* [17], *Camellia sinensis* [18], and *Malus domestica* [19]. These studies provide valuable insights into the evolution and functional diversity of CML proteins across plant species.

Extensive studies have demonstrated the critical roles of CML genes in regulating plant development and stress responses. In *Arabidopsis thaliana*, *AtCML24/25* modulates pollen germination and tube growth [20], while *AtCML9* knockout mutants exhibit enhanced drought and salt tolerance [21]. *AtCML20* negatively regulates ABA signaling, influencing stomatal movement and drought resistance [22], whereas *AtCML39* bidirectionally modulates ABA and GA_3_ signaling pathways [23]. In rice, *OsCML4/5/8/11* participate in osmotic and salt stress responses [24], and *OsCML16* promotes root development and drought tolerance via OsERF48-mediated transcriptional regulation [25]. Horticultural crops also exhibit CML-mediated stress adaptation. Tomato overexpressing *ShCML44* shows improved drought and cold tolerance [26], while *GsCML27* in soybean differentially regulates bicarbonate, salt, and osmotic stresses [27]. In wheat, *TaCML20* enhances drought resistance by modulating water-soluble carbohydrate accumulation [28], and grapevine *VaCML21* improves cold tolerance by activating stress-responsive genes. Additionally, CMLs contribute to plant immunity; *AtCML8* responds to *Pseudomonas syringae* infection [29], and cotton *GhCML11* participates in defense against *Verticillium dahlia* [30]. These findings highlight the functional diversity of *CML* genes across species in developmental and stress adaptation processes.

Potato (*Solanum tuberosum* L.), the world’s fourth most important food crop, plays a vital role in global food security due to its broad adaptability, high productivity, and nutritional value [31,32]. However, drought stress severely constrains potato yield improvement. Recent studies have identified several drought-responsive genes (e.g., *StGA2ox1* [33], *StDRO1* [34], *StPIP1* [35], *StRFP2* [36], and *StMAPK11* [37] that enhance drought tolerance. Despite significant advances in understanding *CML* genes in other species, systematic characterization of the *StCML* gene family, screening of drought-specific responsive members, and clarification of their regulatory mechanisms in drought resistance remain unexplored in potato—addressing this gap is the unique purpose of our study. Here, we performed the first genome-wide identification of 62 *StCML* genes in potato and conducted comprehensive molecular characterization (evolutionary features, chromosomal distribution, and cis-element patterns) using bioinformatics approaches. Through RNA-seq analysis, we revealed their tissue-specific expression patterns and drought-responsive regulation, further screening *StCML50* as a core drought-inducible gene. Furthermore, heterologous overexpression of *StCML50* in Arabidopsis demonstrated its positive role in drought tolerance, and we further clarified its regulatory mechanism via ABA signaling and antioxidant defense systems. This study fills the research blank of potato *StCML* genes in drought response, provides crucial insights into *StCML* gene functions, and establishes a foundation for molecular breeding of drought-resistant potato varieties.

## 2. Results

### 2.1. Identification of StCML Genes

Through comparative genomic analysis utilizing BLAST (https://blast.ncbi.nlm.nih.gov/Blast.cgi), 62 *StCML* genes were identified in the potato genome, demonstrating evolutionary conservation of CML protein sequences across species (Table S1). Bioinformatic characterization revealed substantial variation in StCML protein properties: amino acid lengths spanned 69 (StCML1) to 290 residues (StCML57), with a mean of 158 aa. Corresponding molecular weights ranged from 8.05 to 32.79 kDa (average: 17.82 kDa), while predicted isoelectric points (pI) varied from 3.94 (StCML61) to 9.48 (StCML62), averaging 5.22. Subcellular localization predictions indicated predominant cytoplasmic (22) and nuclear (16) distribution, with minor localization to chloroplasts (11), mitochondria (7), and other compartments.

### 2.2. Structural Characteristics and Chromosomal Distribution of the StCML Family Genes

**Figure 1:**
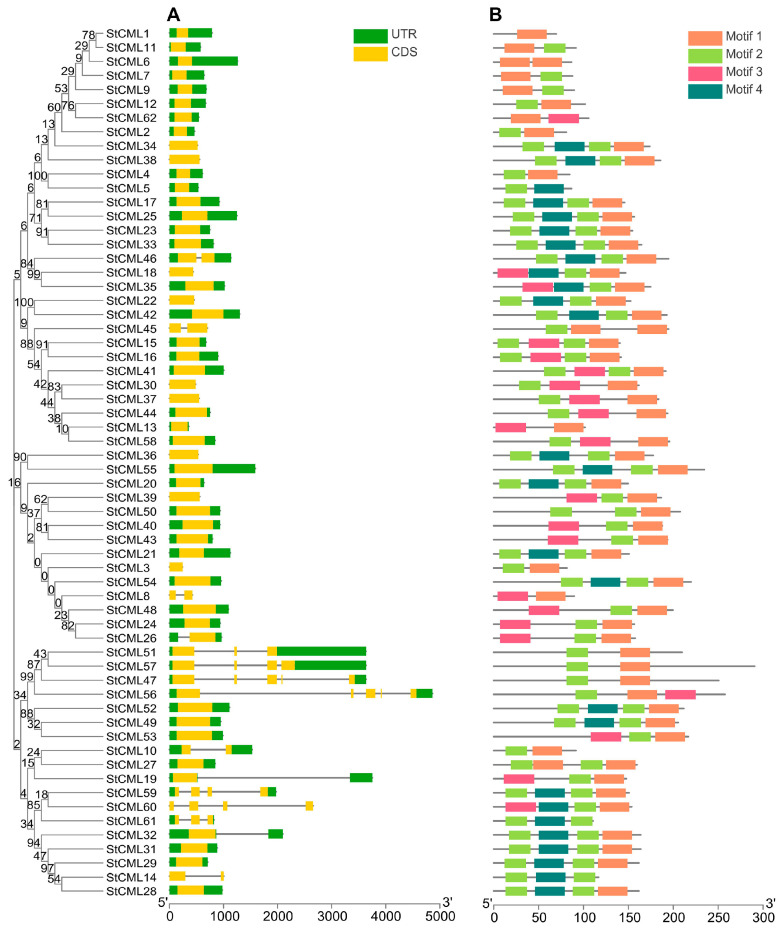
Gene structure and conserved motifs of the *StCML* gene family. (**A**) Schematic gene structure showing 5’ and 3’ UTRs (green boxes), exons (yellow boxes), and introns (black lines). (**B**) Comparison of conserved motifs in StCML proteins.

To elucidate the structural organization of the *StCML* gene family, an exon-intron architecture analysis was performed using genomic DNA sequence alignments (Figure 1A). The results revealed that 75.81% (47/62) of *StCML* genes were intronless, consistent with the compact structure typical of calcium-signaling genes. Among the remaining genes, *StCML47* and *StCML56* exhibited the highest intron count (4 introns each), while other members contained 1–3 introns. This marked heterogeneity in intron distribution suggests that the *StCML* family has undergone frequent intron gain/loss events during evolution, possibly contributing to functional diversification.

Further structural characterization was conducted using MEME motif analysis, which identified four conserved protein motifs (Figure 1B). Among these, Motif1 and Motif2, presumed to represent core functional domains, were present in 87.09% (54/62) of StCML proteins, with only StCML5, StCML14, and StCML61 lacking Motif1. Notably, 91.93% (57/62) of members contained 2–3 motifs, whereas a small subset exhibited either the maximum (4 motifs, 4.84%) or minimum (1 motif, 3.23%) motif counts (Table S2). These variations in motif composition may reflect functional specialization among *StCML* members, potentially influencing their roles in calcium-mediated signaling pathways.

**Figure 2:**
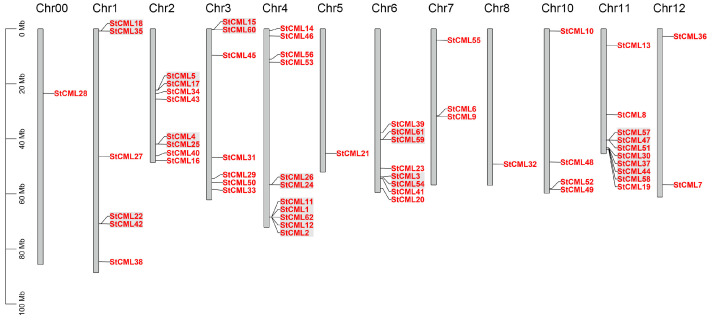
Chromosomal distribution of *StCML* gene family members in potato, with chromosome numbers labeled on the left of each ideogram. The gray background represents *CML* repeat gene pairs.

Chromosomal localization analysis demonstrated that the 62 *StCML* genes were asymmetrically distributed across 12 potato chromosomes (Figure 2). Chromosome 4 harbored the highest gene density (11 genes, 17.74%), whereas Chromosomes 5 and 8 each contained only a single *StCML* gene (1.61%). Intriguingly, 79.03% (49/62) of *StCML* genes were clustered near chromosomal termini, a pattern often associated with evolutionary selection pressure and recombination hotspots. Additionally, 11 allelic gene pairs were identified, likely arising from tandem duplication events, suggesting that local genomic duplications have significantly contributed to the expansion of the *StCML* family. These findings provide insights into the structural diversity, evolutionary dynamics, and potential functional divergence of *StCML* genes in potato.

### 2.3. Phylogenetic Analysis Divided StCML into 7 Evolutionary Branches

**Figure 3:**
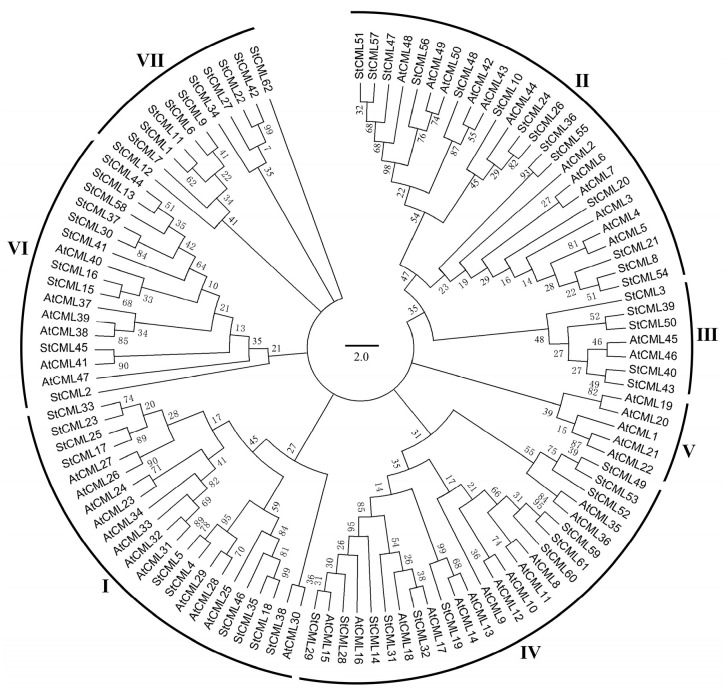
Phylogenetic analysis of *Arabidopsis* and potato using MEGA 11.0. The ML tree was constructed with 1000 bootstrap replicates (default parameters). I–VII represent the seven subgroups of *StCML* family genes.

A maximum-likelihood phylogenetic tree was constructed using 112 CML proteins, comprising 62 StCMLs from potato and 50 AtCMLs from *Arabidopsis* (Figure 3). The analysis revealed seven distinct evolutionary clades (I-VII), demonstrating differential distribution patterns between the two species. Notably, potato StCMLs showed preferential clustering in Clade VI (22 members, 35.48% of total StCMLs), while *Arabidopsis* AtCMLs were predominantly localized in Clades I and IV (10 members each, 20% of total AtCMLs). Comparative analysis indicated that StCMLs outnumbered AtCMLs (62 vs. 50) and were present in all clades, potentially reflecting genome duplication events in the tetraploid potato. The clade-specific distribution varied significantly (Clades I-VII contained 16, 21, 13, 18, 11, 28, and 5 proteins, respectively), suggesting potential functional divergence among different evolutionary lineages.

### 2.4. Intraspecific/Interspecific Analysis of the StCML Gene Family

**Figure 4:**
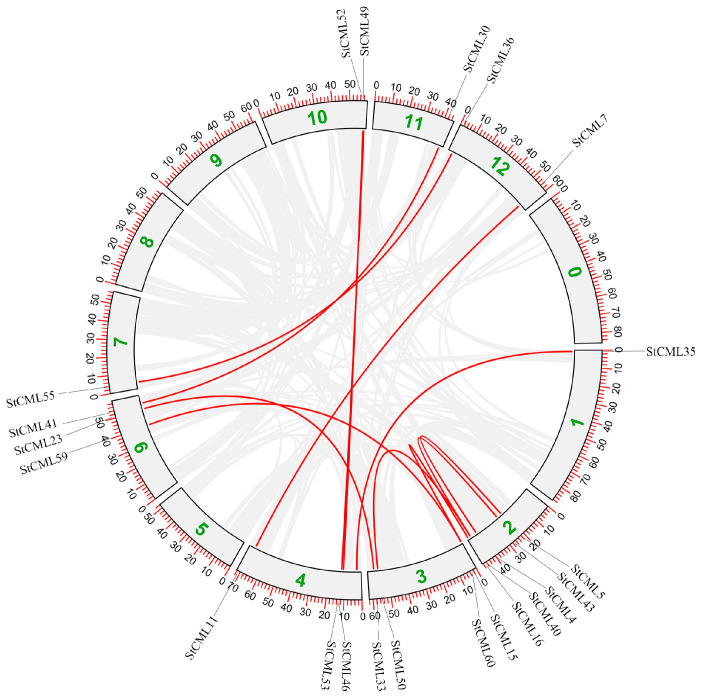
Synteny analysis of *StCML* genes in potato chromosomes using MCScanX (1.0.0) and TBtools. Gray lines show genome-wide duplication blocks; red lines highlight syntenic CML gene pairs. Chromosome numbers appear as perimeter-positioned boxes. Green numbers represent different chromosomes of potatoes.

To investigate the evolutionary mechanisms driving *StCML* family expansion, intra-species synteny analysis was performed (Figure 4). Tandem and segmental duplications were classified based on established genomic criteria: tandem duplications were defined as two or more homologous *StCML* genes located within a 100 kb genomic window without intervening non-homologous genes, and sharing > 80% sequence identity; segmental duplications were identified as homologous gene pairs mapped to non-adjacent chromosomal regions, with collinear arrangement of flanking genes (≥3 syntenic gene pairs in the surrounding 500 kb interval) and consistent gene orientation. The results demonstrate that both duplication types contributed significantly to gene family diversification. Specifically, five tandem duplication pairs were identified across multiple chromosomal regions, indicating recurrent local duplication events. Furthermore, seven large-scale segmental duplications were detected, facilitating widespread genomic expansion. These findings collectively suggest that *StCML* family evolution has been predominantly driven by genomic duplication events, including localized tandem repeats and chromosomal segmental duplications, which have collectively enabled functional diversification through gene copy number variation.

Cross-species synteny analysis revealed evolutionary relationships between potato *StCML* genes and five species (Figure S1). The highest number of homologous genes was observed with tomato (*Solanum lycopersicum*) and soybean (*Glycine max*) (34 each), followed by grape (*Vitis vinifera*) and sunflower (*Helianthus annuus*) (27 each), while tartary buckwheat (*Fagopyrum tataricum*) showed the fewest homologs (12). Corresponding syntenic gene pairs numbered 66, 67, 18, 31, and 54, respectively (Table S3). These findings indicate both extensive lineage-specific duplication events and conserved evolutionary relationships among eudicot *CML* genes, with particularly strong conservation between solanaceous species.

### 2.5. Analysis of Cis-Acting Elements of the Potato CML Family

Comprehensive examination of cis-acting elements within the 2-kb promoter regions of *StCML* genes (Figure S2) revealed four predominant functional categories: hormonal regulation, stress response, metabolic control, and light responsiveness. Stress-related analysis demonstrated that ABA-responsive elements (ABRE; *n* = 122) predominated among hormone-responsive elements, followed by MeJA- (CGTCA-motif; 66), salicylic acid- (TCA-element; 41), auxin- (TGA-element; 17), and gibberellin-responsive elements (GARE-motif; 17). Notably, light-responsive (TCT-motif; 52), drought-inducible (MBS; 43), low-temperature-responsive (LTR; 32), and endosperm-specific elements (GCN4-motif; 16) were identified, while flavonoid biosynthesis-related elements (MBSI; 6) were least abundant. This regulatory landscape suggests *StCML* genes are potentially modulated by diverse environmental and developmental cues.

### 2.6. Expression Patterns of StCML Genes in Different Potato Tissues

Transcriptome analysis across 14 potato tissues revealed distinct expression patterns among *StCML* family members (Figure S3). Eight genes (including *StCML52/53*) exhibited constitutive high expression across all tissues, while seven members (e.g., *StCML18/22*) showed no detectable expression. The remaining genes displayed tissue-specific expression profiles: *StCML2/11* demonstrated root-predominant expression, whereas *StCML4/43* were preferentially expressed in reproductive tissues (flowers and stamens). These results suggest functional diversification within the *StCML* family, with subsets of genes potentially involved in tissue-specific physiological processes while others may maintain fundamental cellular functions.

### 2.7. Response of StCML Genes to Drought Stress

**Figure 5:**
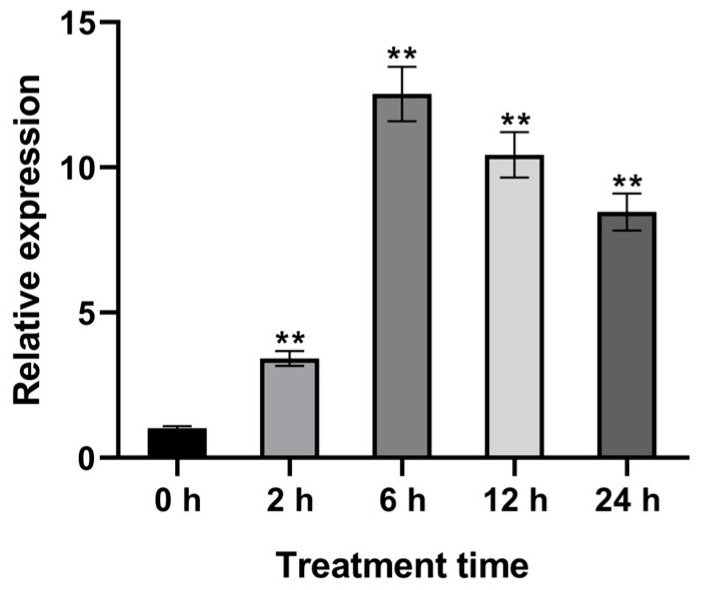
Transcriptomic analysis of *StCML50* gene under drought stress. Three-week-old in vitro seedlings received 200 mM mannitol stress in 1/2 MS medium, the expression was detected at 0, 2, 6, 12, and 24 h post-treatment. Asterisks indicate significant differences (** *p* < 0.01).

Promoter analysis of *StCML* genes suggested their potential involvement in diverse hormonal and abiotic stress responses. Given our research focus on potato drought resistance, we systematically investigated *StCML* expression patterns under water deficit conditions (Figure S4). Transcriptomic profiling revealed three distinct response categories: (1) Genes exhibiting baseline expression with minimal stress-responsive fluctuations (e.g., *StCML2*, *StCML4*); (2) Constitutively expressed genes showing limited stress responsiveness (e.g., *StCML52*, *StCML56*); and (3) Highly stress-responsive genes demonstrating dramatic induction, exemplified by *StCML50* (showing 51–54 fold up-regulation post-stress) and *StCML58*. This differential regulation suggests functional specialization within the *StCML* family, with specific members potentially playing pivotal roles in drought stress adaptation. The marked induction of certain *StCML* genes (particularly *StCML50*) indicates their potential as key regulators in potato’s drought response network, warranting further functional characterization (Figure 5).

### 2.8. StCML50 Enhances the Germination Rate of Transgenic Plants Under Drought Stress

**Figure 6:**
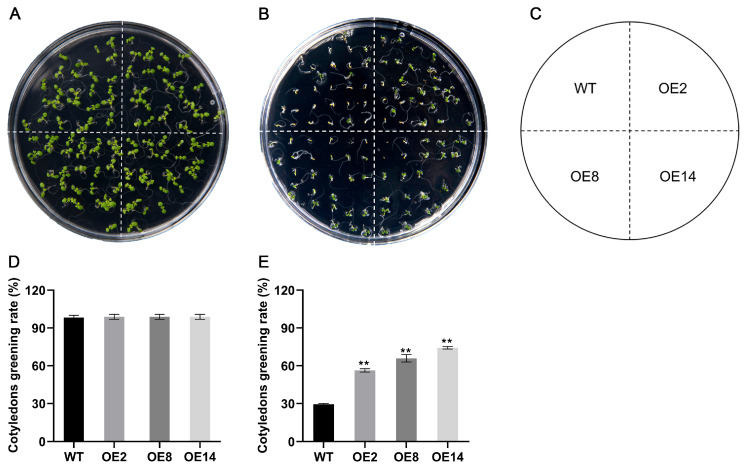
The cotyledon greening rate of *StCML50* transgenic and WT lines under 200 mM mannitol stress. (**A**–**C**) Phenotypes under control and stress conditions. (**D**,**E**) Statistics on the cotyledon greening rate under normal and stress conditions. ** indicates significant difference at *p* < 0.01 level.

**Figure 7:**
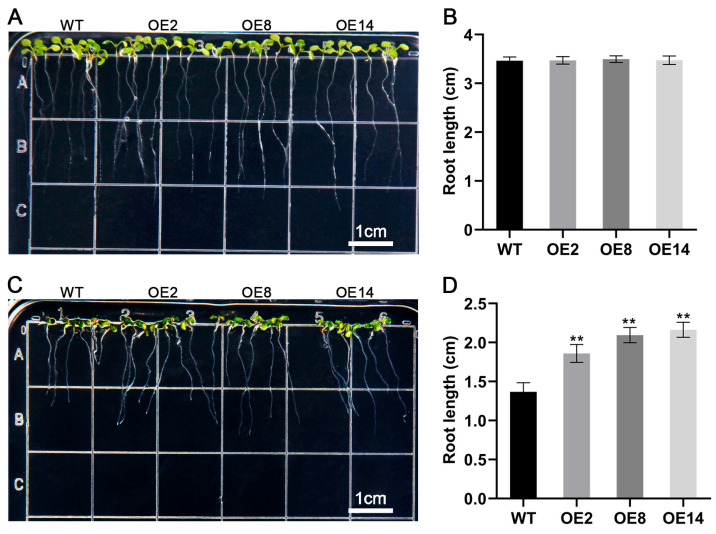
Root phenotypes of *StCML50* transgenic and WT lines under 200 mM mannitol stress. (**A**,**B**) Root morphology and quantification under control conditions. (**C**,**D**) Root characteristics and statistical analysis under drought stress. Asterisks indicate significant differences (** *p* < 0.01).

Given the marked up-regulation of *StCML50* under water-deficit conditions, we generated *StCML50*-overexpressing transgenic *Arabidopsis* lines and selected three homozygous T3 lines (OE2, OE8, and OE14) with high transgenic expression for phenotypic analysis (Figure S5). Under control conditions (1/2 MS medium), no significant differences were observed between transgenic lines and wild-type (WT) plants in cotyledon greening or primary root elongation. However, under drought stress (200 mM mannitol), transgenic lines exhibited significantly higher cotyledon greening rates (Figure 6) and longer primary roots (Figure 7) compared to WT, though both genotype showed stress-induced growth inhibition. These results demonstrate that *StCML50* enhances tolerance to water-deficit stress without altering developmental phenotype under non-stress conditions, supporting its role as a positive regulator of drought adaptation.

### 2.9. StCML50 Improves the Resistance of Transgenic Plants Against Drought Stress

**Figure 8:**
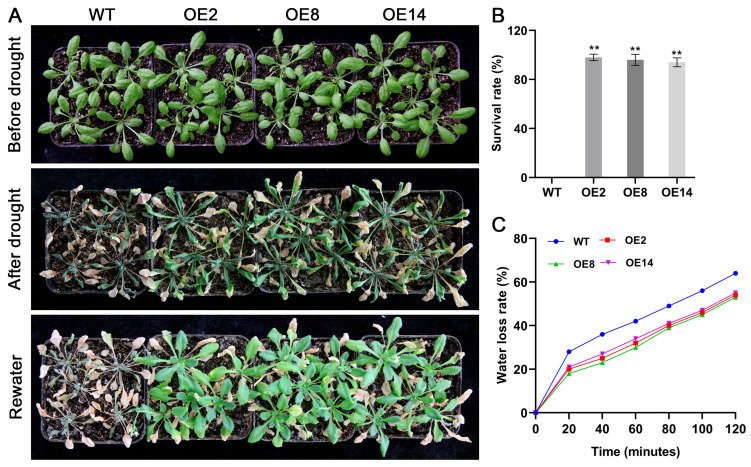
*StCML50* overexpression improves drought tolerance in transgenic plants. (**A**) Phenotypic comparison of transgenic lines and WT under drought. (**B**) Survival rate and (**C**) water loss quantification after 19-day drought (initiated at 15 DPG) and 4-day rehydration. Data show mean ± SD of three biological replicates. ** *p* < 0.01 (*t*-test).

To further validate the drought-responsive function of *StCML50*, we subjected 3-week-old soil-grown transgenic and WT plants to water deficit stress. Under normal irrigation, no phenotypic differences were observed between genotype (Figure 8). Following 15 days of stress, both WT and transgenic plants exhibited leaf curling and wilting; however, transgenic lines retained partial leaf greenness while WT leaves completely bleached. Post-stress measurements revealed significantly higher fresh weight in transgenic plants. After 4 days of re-watering, transgenic plants demonstrated superior recovery, with higher leaf re-greening capacity and survival rates compared to WT. Although fresh weights were comparable immediately after stress, transgenic plants exhibited significantly greater biomass recovery post-rewatering. These results confirmed that *StCML50* enhances drought tolerance by improving water retention and recovery capacity.

### 2.10. Physiological and Biochemical Characteristics of Transgenic Arabidopsis Under Drought Treatment

**Figure 9:**
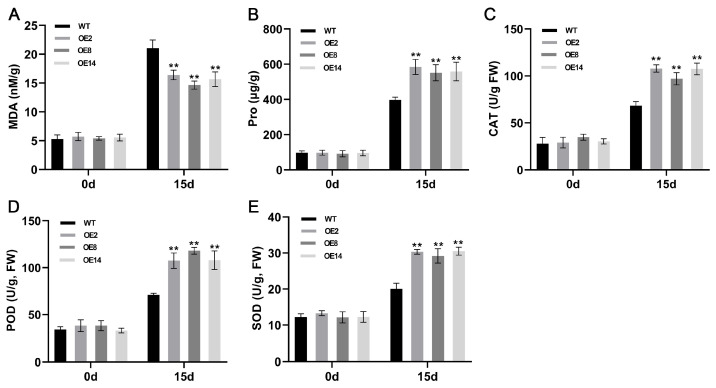
Physiological parameters under drought stress. (**A**–**E**) MDA, proline, CAT, POD, and SOD activities, respectively. Asterisks denote statistically significant differential expression between transgenic and wild-type plants (** *p* < 0.01, Student’s *t*-test).

Physiological and biochemical analyses were conducted to evaluate drought resistance mechanisms in StCML50-overexpressing lines. Under control conditions, no significant differences were observed in MDA content or antioxidant enzyme activities between transgenic and WT plants. However, under water deficit stress, transgenic lines exhibited: (1) 26.11% lower MDA accumulation, indicating reduced membrane damage; (2) 1.42-fold higher proline content, suggesting enhanced osmotic adjustment; and (3) 20.12% slower water loss rate, demonstrating improved water retention capacity. Antioxidant enzyme assays revealed significantly elevated activities of SOD (1.49-fold), POD (1.56-fold), and CAT (1.52-fold) in stressed transgenic plants compared to WT (Figure 9). These results demonstrated that *StCML50* confers drought tolerance through multiple mechanisms, including enhanced antioxidant defense, osmotic regulation, and cellular membrane protection.

### 2.11. StCML50 Increases the Expression Level of Stress Response Genes

**Figure 10:**
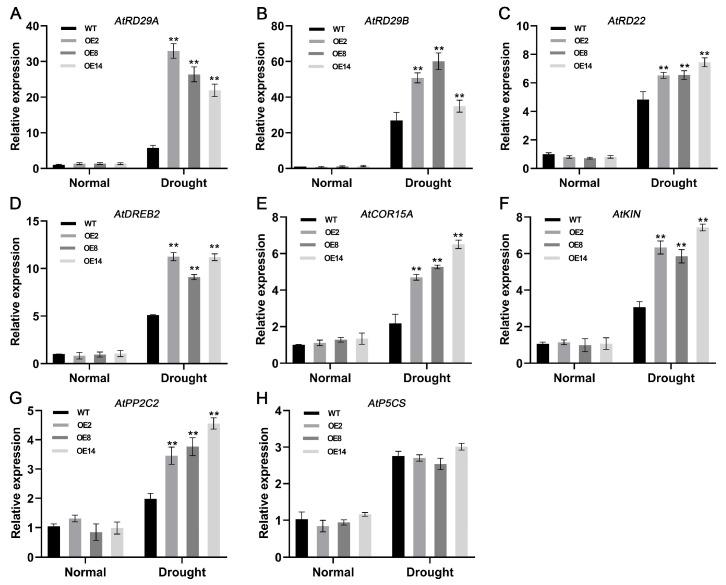
Stress-responsive gene expression in transgenic vs. WT plants. Stem segments from uniform transgenic lines were transferred to basal MS or 200 mM mannitol-MS medium. After 30 days, aerial tissues were collected for qRT-PCR. Gene expression profiles: (**A**) *AtRD29A*, (**B**) *AtRD29B*, (**C**) *AtRD22*, (**D**) *AtDREB2*, (**E**) *AtCOR15A*, (**F**) *AtKIN*, (**G**) *AtPP2C2*, and (**H**) *AtP5CS*. Asterisks indicate significant differences (** *p* < 0.01, *t*-test).

To investigate the molecular mechanism of *StCML50* in drought stress more deeply, the expression levels of several genes in transgenic and WT plants were examined under normal and drought conditions (Figure 10). Under normal growth conditions, the expression levels of stress-responsive genes in *StCML50* overexpressing and wild-type plants were essentially the same. Under drought stress, these genes were differentially up-regulated in *StCML50* overexpressing lines and wild-type plants. Among them, drought stress-related genes such as *RD29A*, *RD29B*, *RD22*, *DREB*, and *PP2C* were significantly elevated in Arabidopsis overexpressing *StCML50*.

### 2.12. StCML50 Enhances ABA Sensitivity in Transgenic Arabidopsis

**Figure 11:**
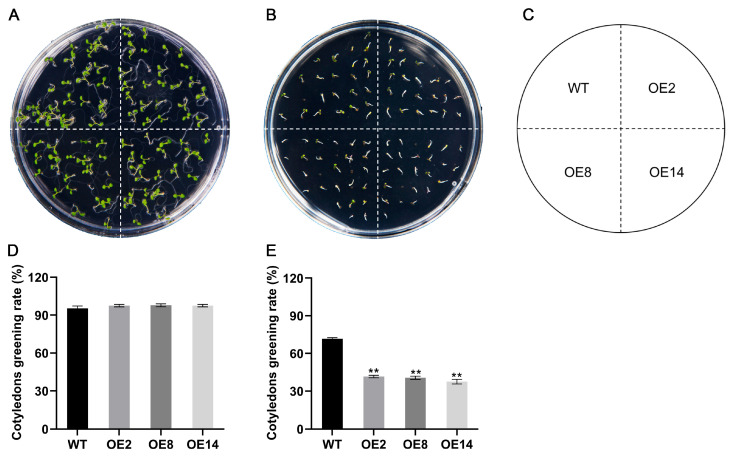
Cotyledon greening rate of *StCML50* transgenic and WT lines under ABA stress. (**A**–**C**) Phenotypes under control and stress conditions. (**D**,**E**) Statistics on the cotyledon greening rate under normal and stress conditions. Asterisks indicate significant differences (** *p* < 0.01, *t*-test).

**Figure 12:**
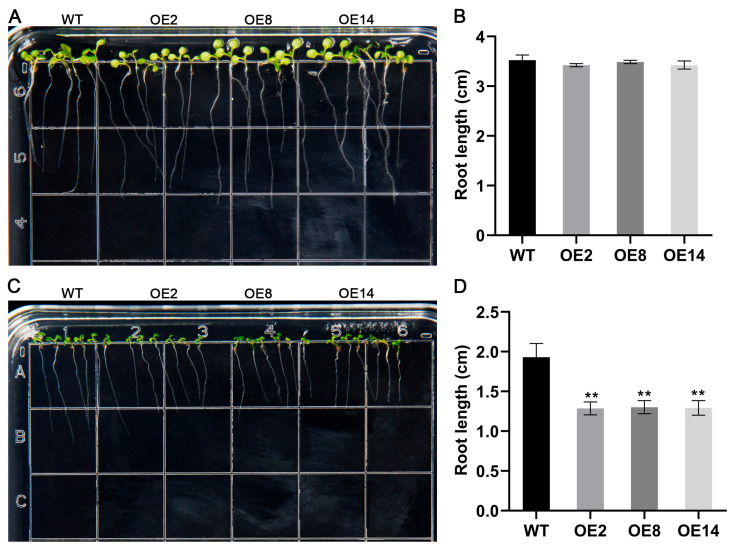
Root phenotypes of *StCML50* transgenic and WT lines under ABA treatment. (**A**,**B**) Root morphology and quantification under control conditions. (**C**,**D**) Root characteristics and statistical analysis under ABA treatment. Asterisks indicate significant differences (** *p* < 0.01, *t*-test).

To assess the involvement of *StCML50* in ABA signaling, we examined the ABA sensitivity of *StCML50*-overexpressing lines using germination and root elongation assays. Under control conditions, no phenotypic differences were observed between transgenic and WT plants. However, on ABA-supplemented medium, transgenic lines exhibited significantly stronger inhibition of cotyledon greening (Figure 11) and root elongation (Figure 12) compared to WT, indicating enhanced ABA sensitivity. Furthermore, qPCR analysis revealed elevated expression of ABA-responsive genes (*AtRD29A*, *AtRD29B*, *AtPP2CA*, and *AtRD22*) in *StCML50* transgenic plants under drought stress. These findings demonstrated that *StCML50* potentiates ABA signaling, suggesting its role in regulating ABA-dependent stress responses. The increased ABA sensitivity and upregulation of ABA-related genes in transgenic plants imply that *StCML50* may function as a positive modulator of ABA-mediated drought adaptation.

## 3. Discussion

As a crucial secondary messenger, calcium (Ca^2+^) regulates diverse physiological processes in plants through complex signaling networks [4]. Ca^2+^ sensor proteins, including CML proteins, decode Ca^2+^ signals by binding Ca^2+^ via EF-hand motifs and transducing these signals to downstream targets [38]. Unlike other Ca^2+^ sensors, plant-specific CMLs exclusively contain EF-hand domains without additional functional modules [39]. Our genome-wide analysis identified 62 *StCML* genes in potato exhibiting substantial structural diversity, as evidenced by variations in protein length, molecular weight, and isoelectric points. This structural heterogeneity suggests functional specialization among *StCML* family members in mediating Ca^2+^-dependent signaling pathways.

The potato genome encodes *62 StCML* genes, a number comparable to grapevine (62 *VviCMLs*) but distinct from other species (Arabidopsis: 50 *AtCMLs* [40]; apple: 58 *MdCMLs* [41]; B. napus: 168 *BnaCMLs* [13]), suggesting lineage-specific expansion patterns. Structural analysis revealed StCML proteins contain 1–4 EF-hand domains, consistent with typical Ca^2+^-binding capacity. Phylogenetic classification grouped StCMLs into 7 clades, mirroring wheat’s organization [41] but exhibiting fewer subgroups than Nelumbo nucifera (12 clades) [42]. Notably, clade VI represented the largest group (*n* = 12), while co-clustering with AtCMLs implied conserved functional roles [43]. Genomic architecture analysis showed 75.81% (47/62) of StCMLs were intronless, a prevalence comparable to Arabidopsis (74%) [44] and rice (75%) [11] but exceeding tomato (59.62%). This structural conservation suggests evolutionary selection for rapid transcriptional responses, as intron-poor genes typically exhibit faster expression kinetics [45,46]—a critical advantage for Ca^2+^ sensor genes that need to rapidly decode transient Ca^2+^ signals during stress exposure. The higher intronless ratio in potato compared to tomato may reflect lineage-specific optimization of stress response efficiency, aligning with potato’s adaptation to diverse growing environments. The observed variation in intron content may result from partial duplication events influencing gene structure. These findings collectively demonstrate that while *CML* genes maintain core structural features across plants, their genomic organization shows both conserved and divergent evolutionary trajectories, potentially contributing to functional specialization in calcium signaling pathways.

Chromosomal localization results showed that *StCMLs* were unevenly distributed on all 12 chromosomes, and the *StCML* gene family contained 28 (45.16%) tandem duplication genes. Arabidopsis contains 11 (22%) tandem duplication genes [43]. In comparison the percentage of gene duplication was higher in potato. Gene duplication analysis further confirmed that there were more segmental duplications (7 pairs) than tandem duplications (5 pairs) as the main driver of *StCML* evolution. Specific expression of genes is closely linked to cis-acting elements in their promoter regions. Previous studies have shown that certain *CML* genes with specific cis-acting elements are involved in hormonal or abiotic stress responses [47]. For example, the promoter region of the *AtCML9* gene is enriched in ABRE and GT1-box elements, and its expression was significantly induced by salinity, drought, and aba treatments [21]. *StCMLs* contain a variety of cis-acting elements related to hormones, growth and development, and stress. *StCMLs* contain cis-acting elements related to ABA (ABRE) and drought stress (MBS), suggesting that *StCMLs* may be extensively involved in the ABA pathway to regulate drought stress.

Extensive studies have demonstrated the critical involvement of CML proteins in plant development and stress adaptation. In Arabidopsis, *AtCML39* regulates reproductive development, with knockout mutants exhibiting shortened siliques and reduced seed set [23]. Rice *OsCML4* enhances drought tolerance through ROS scavenging and activation of stress-responsive genes [48]. Our expression profiling revealed tissue-specific patterns among *StCML* genes, with *StCML11* and *StCML2* showing root-predominant expression. Notably, *StCML50* exhibited dramatic drought induction (51–54 fold increase) in potato cultivars—a strong indication of its potential role in potato drought response, though direct functional evidence in potato remains to be established. Given the established roles of ABA and MeJA as key drought signaling molecules, we identified 24 ABA-responsive *StCML* genes, suggesting their participation in hormonal stress response pathways. These findings collectively highlight the functional diversification of *CML* family members in both developmental processes and abiotic stress adaptation, with particular relevance to drought response mechanisms in potato.

To elucidate the molecular mechanisms underlying drought tolerance mediated by *StCML* genes, we focused our investigation on *StCML50*, a candidate gene showing remarkable induction under water deficit conditions. Transgenic Arabidopsis thaliana lines overexpressing *StCML50* were established and subjected to comprehensive phenotypic and physiological analyses under drought stress conditions. Our results demonstrated that *StCML50* overexpression conferred significant drought tolerance, as evidenced by multiple physiological parameters. Transgenic plants exhibited enhanced cotyledon greening rates, maintained greater root elongation capacity, and showed significantly improved survival rates under water deficit conditions compared to wild-type controls. These observations strongly suggest that *StCML50* plays a crucial role in plant drought adaptation mechanisms. The enhanced drought tolerance observed in *StCML50*-overexpressing lines appears to be mediated through multiple protective mechanisms. First, we observed a significant elevation in the activity of key antioxidant enzymes, including superoxide dismutase (SOD), peroxidase (POD), and catalase (CAT). These enzymes collectively maintain cellular redox homeostasis by scavenging reactive oxygen species (ROS) that accumulate under stress conditions [49]. The increased antioxidant capacity in transgenic plants likely contributes to reduced oxidative damage, as supported by our observation of lower malondialdehyde (MDA) content—a reliable biomarker of membrane lipid peroxidation [50,51]. Furthermore, *StCML50* overexpression led to enhanced osmotic adjustment capacity, as indicated by elevated proline accumulation. As a compatible osmolyte, proline plays dual roles in maintaining cellular turgor pressure and protecting macromolecular structures under dehydration stress [52]. The coordinated upregulation of both antioxidant defenses and osmoprotectant accumulation in *StCML50*-overexpressing plants provides a comprehensive protective network against drought-induced damage. Notably, the reduced MDA content in transgenic lines suggests that *StCML50* helps maintain membrane integrity under stress conditions. This finding is particularly significant as membrane stability is a key determinant of plant survival under water deficit [53]. The observed physiological improvements (enhanced antioxidant capacity, osmotic adjustment, and membrane stability) collectively explain the superior drought tolerance phenotype of *StCML50* transgenic plants. These results position *StCML50* as a promising candidate for genetic improvement of drought tolerance in crops. The multifaceted protective mechanisms mediated by *StCML50*—encompassing both ROS scavenging and osmotic adjustment—suggest its potential as a master regulator of stress responses. Future studies should investigate the downstream signaling pathways and target genes regulated by *StCML50* to fully elucidate its role in drought adaptation networks. While this study successfully demonstrated that *StCML50* enhances drought tolerance through physiological adjustments (e.g., osmotic regulation and antioxidant defense), the precise molecular mechanism remains to be fully elucidated. Future studies will focus on identifying the downstream interacting partners of *StCML50* using yeast two-hybrid (Y2H) and bimolecular fluorescence complementation (BiFC) assays to map the specific signaling pathway.

## 4. Materials and Methods

### 4.1. Plant Materials

The potato cultivar ‘Qingshu 9’ (drought-resistant), along with *Arabidopsis thaliana* ecotype ‘Columbia-0’, were provided by Gansu Agricultural University. ‘Qingshu 9’ is widely cultivated in Northwest China and exhibits significant drought tolerance, making it an ideal genotype for studying stress-responsive genes. Both species were cultivated in growth chambers under controlled conditions (16/8 h light/dark cycle, photosynthetically active radiation (PAR) of 250 μmol photons m^−2^ s^−1^, temperature of 22 ± 2 °C, and relative humidity (RH) of 60%). Potato plantlets were maintained in vitro on full-strength MS medium, while *A. thaliana* seeds were processed following the standardized procedure below: first stratified at 4 °C for 3 days, then surface-sterilized by sequential immersion in 75% ethanol for 1 min and 10% sodium hypochlorite for 1 min, followed by five rinses with sterile water to remove residual disinfectants. The sterilized seeds were either sown on 1/2 MS medium (pH adjusted to 5.8) or directly planted into a sterilized growth substrate (peat:vermiculite = 3:1, *v*/*v*) for subsequent cultivation.

### 4.2. Identification of Potato StCML Genes

Genomic data (DM v6.1 genome assembly, GFF annotation file) for potato (FASTA/GFF formats) were acquired from the Potato Genomics Resource (http://spuddb.uga.edu, accessed on 5 March 2024), while 50 typical AtCML protein sequences containing complete EF-hand domains were retrieved from TAIR (https://arabidopsis.org, accessed on 6 March 2024). StCML candidates were identified via dual BLASTP searches in TBtools (bit-score ≥ 100, e-value ≤ 1 × 10^−10^). Protein validation included conserved domain analysis (CD-Search, NCBI) and motif identification (MEME Suite). Verified StCML members were systematically annotated following established nomenclature conventions for plant *CML* gene familie.

### 4.3. Structural and Chromosomal Analysis of Potato StCML Genes

The conserved motifs of StCML proteins were analyzed using the MEME online tool (v5.4.1, http://meme-suite.org/tools/meme, accessed on 10 March 2024) with the following parameter settings: maximum number of motifs = 10, and motif width ranging from 6 to 50 amino acids. Conserved domains were identified via the NCBI Batch CD-Search tool (https://www.ncbi.nlm.nih.gov/Structure/bwrpsb/bwrpsb.cgi, accessed on 3 April 2024), with a focus on the presence of EF-hand calcium-binding domains. The results of motif and domain analyses, along with the intron/exon structures and chromosomal distribution of StCML members, were all visualized using TBtools software (v1.09876).

### 4.4. Phylogenetic Analysis of Potato StCML Proteins

CML protein sequences from *Arabidopsis* and potato were downloaded from Ensembl Plants databases (http://plants.ensembl.org/, accessed on 21 April 2024). All sequences were aligned using the ClustalW algorithm implemented in MEGA software (11.0). A phylogenetic tree was constructed via the neighbor-joining (NJ) method with the following parameters: Poisson model, pairwise deletion of gaps, and 1000 bootstrap replicates to assess node reliability. The evolutionary relationships of StCML proteins were further visualized using the EvolView online platform (https://evolgenius.info/evolview-v2/#login, accessed on 21 April 2024).

### 4.5. Intra- and Interspecies Synteny Analysis of StCML Genes

Genomic synteny analysis was performed using TBtools software, first through self-alignment of potato reference genome and annotation (GFF) files to identify paralogous *StCML* gene pairs. Comparative genomic analyses were then conducted by aligning potato sequences with those of six representative species (*Arabidopsis*, tomato, soybean, grape, tartary buckwheat, and sunflower) retrieved from EnsemblPlants. All syntenic relationships were visualized using TBtools’ built-in functions.

### 4.6. Cis-Acting Element Analysis of Potato StCML Genes

The 2000 bp promoter regions (upstream of the translation start site, ATG) of StCML genes were extracted using TBtools software. If another gene was present within the 2000 bp upstream region, the sequence was truncated to the downstream edge of the adjacent gene to avoid overlapping with non-promoter regions. Cis-regulatory elements were analyzed via the PlantCARE online tool (v5.0, http://bioinformatics.psb.ugent.be/webtools/plantcare/html/, accessed on 25 June 2024). Additionally, predicted transcription factor binding sites (TFBSs) were identified using the PlantRegMap database (https://ngdc.cncb.ac.cn/databasecommons/database/id/6966, accessed on 25 April 2024) with a confidence threshold of 0.8, and Solanum tuberosum was set as the reference species. The distribution of cis-acting elements was visualized using TBtools.

### 4.7. Construction and Stress Treatment of Genetically Modified Materials

The full-length coding sequence (CDS) of *StCML50* was PCR-amplified using gene-specific primers and cloned into the binary vector pCAMBIA1304 (driven by the CaMV 35S promoter), generating the recombinant overexpression plasmid pCAMBIA1304-StCML50. The pCAMBIA1304 vector carries the *hpt* gene (hygromycin phosphotransferase) as a plant selectable marker [54]. We utilized *Arabidopsis* as a heterologous system for rapid proof-of-concept functional verification. This construct was introduced into wild-type *Arabidopsis thaliana* (ecotype Col-0) via Agrobacterium-mediated floral dip transformation [55], using *Agrobacterium* tumefaciens strain GV3101. Primary transformants (T1 generation) were selected on 1/2MS medium supplemented with 50 mg/L hygromycin. Surviving seedlings were transplanted to sterilized soil (peat:vermiculite = 3:1, *v*/*v*) and grown under the controlled conditions described in Section 2.1. Putative transgenic lines were validated by PCR amplification of the *StCML50* CDS (using genomic DNA as template). T3 homozygous overexpressing lines (with stable inheritance) were established for subsequent functional characterization.

Seeds of transgenic lines and wild-type (WT) *A. thaliana* were processed following the standardized procedure described in Section 2.1; after sterilization and air-drying, the seeds were plated on 1/2 MS medium (pH adjusted to 5.8) supplemented with 0 mM (control) or 200 mM mannitol for osmotic stress tolerance assay. Germination rate (defined as radicle emergence ≥ 2 mm) and primary root length were recorded after five days of cultivation under the conditions specified in Section 2.1, with three biological replicates (*n* = 3) and 50 seeds per replicate. For drought stress assay, transgenic and WT seeds were sown in the sterilized growth substrate (peat:vermiculite = 3:1, *v*/*v*) and grown under the conditions described in Section 2.1 for three weeks, followed by 15 days of water withholding to impose drought stress and subsequent 4 days of rehydration. Each drought treatment included three biological replicates (*n* = 3) with 10 plants per replicate.

Physiological responses were assessed 4 day after rehydration by measuring the following indices: survival rate (percentage of plants with green and turgid leaves after rehydration); fresh weight (FW) of aboveground tissues (weighed with an analytical balance after blotting surface moisture); relative water content (RWC) of rosette leaves (calculated using the formula: RWC (%) = [(FW − DW)/(TW − DW)] × 100%, where FW = fresh weight, TW = turgid weight, DW = dry weight); proline (Pro) content (acidic ninhydrin method); malondialdehyde (MDA) content (thiobarbituric acid colorimetric method); superoxide dismutase (SOD) activity (nitroblue tetrazolium method); peroxidase (POD) activity (guaiacol method); and catalase (CAT) activity (ultraviolet absorption method). All physiological indices were determined with three biological replicates, and crude enzyme extracts for antioxidant enzyme activity assays were freshly prepared.

### 4.8. Expression Patterns of StCMLs in Potato

Based on Illumina RNA-seq data retrieved from SpudDB (https://spuddb.uga.edu/, accessed on 25 July 2024), a genomic resource hosting data from the International Potato Genome Sequencing Consortium (PGSC), this study analyzed the expression patterns of StCML genes in 14 DM potato tissues (stamens, leaves, stolons, shoots, tubers, roots, callus, carpels, petioles, petals, flowers, sepals, immature fruit, mature fruit), and within in-vitro whole plant exposed to abiotic stresses (heat treatment at 35 °C; salt treatment with 150 mM NaCl; mannitol-mediated drought stress with 260 mM mannitol) and hormone treatments (abscisic acid-ABA: 50 μM, 24 h; benzylaminopurine-BAP: 10 μM, 24 h; gibberellic acid-GA_3_: 50 μM, 24 h; indole acetic acid-IAA: 10 μM, 24 h). The heatmap was plotted with the TBTools program.

Gene expression profiles under various stress conditions were analyzed via quantitative real-time PCR (qRT-PCR). Specific primers were designed using Primer Premier 7, with potato *EF1α* serving as the internal control, as it has been validated as a stable reference gene in potato under abiotic stress conditions [56]. Reactions (10 μL) comprised 5 μL PrimeSTAR^®^ Premix Ex Taq™ (Probe qPCR) (Takara Bio Inc., Kusatsu, Japan), 1 μL primers, and 4 μL ddH_2_O. Thermal cycling conditions: 95 °C for 3 min; 45 cycles of 95 °C for 5 s and 60 °C for 30 s. Relative expression levels were quantified using the 2^−ΔΔCT^ method [57]. All qRT-PCR reactions were performed with three biological replicates and three technical replicates. All primer sequences are detailed in Supplementary Table S4.

### 4.9. Statistical Analysis

Data obtained from three independent biological replicates (each representing distinct plant individuals) were presented as means ± standard deviation (SD). Statistical analyses were performed using SigmaPlot 10.1 software. Two-tailed Student’s *t*-test was used for comparisons between two groups, while one-way analysis of variance (ANOVA) followed by Tukey’s post-hoc test was applied for multiple-group comparisons. Differences were considered statistically significant at *p* < 0.05 and highly significant at *p* < 0.01.

## 5. Conclusions

In summary, this study identified 62 *StCML* genes in potato, with expression analyses implying their potential roles in development and stress responses. Functional characterization in the heterologous *Arabidopsis* system revealed that *StCML50* enhances drought tolerance, as transgenic lines overexpressing *StCML50* exhibited improved stress resilience. Mechanistically, this effect may be mediated by *StCML50*-upregulated ABA signaling, proline biosynthesis, ROS scavenging, and drought-responsive pathways in *Arabidopsis*. These findings advance our understanding of *StCML* gene functional potential and highlight *StCML50* as a promising candidate for further validation in potato. Future reverse genetics studies in potato are needed to confirm *StCML50*’s direct role in drought tolerance, laying a more solid foundation for breeding stress-resistant cultivars.

## Data Availability

The original contributions presented in this study are included in the article/Supplementary Material. Further inquiries can be directed to the corresponding authors.

## Supplementary Materials

The following supporting information can be downloaded at: https://www.mdpi.com/article/10.3390/plants15030417/s1. Figure S1. Synteny relationships of *CML* genes between potato and five other plants species. Gray lines in the background represent genome-wide collinear blocks across the genomes of the six species, while red lines specifically highlight syntenic *CML* gene pairs between potato and the other five plants; Figure S2. Analysis of cis-regulatory elements in the *StCML* gene family. The distribution of predicted cis-regulatory elements within the 2.0-kb upstream promoter regions of *StCML* family genes was analyzed, with a specific focus on elements associated with environmental stress responses; Figure S3. Tissue-specific expression profiling of *StCML* gene family members. Hierarchical clustering analysis was performed on the transcript abundance of *StCML* genes across multiple potato tissues, and results are presented as a heatmap. Expression levels were normalized via log_2_(FPKM + 1), where FPKM denotes fragments per kilobase of transcript per million mapped reads. The color gradient represents relative expression intensities, with a color scale bar denoting relative fold-change values; Figure S4. Transcriptomic analysis of *StCML* genes in potato under drought stress. Expression profiles (normalized as log_2_[FPKM+1]) were generated for the drought-tolerant potato cultivar ‘Qingshu NO.9’ (QS9). Three-week-old in vitro-grown potato seedlings were treated with 200 mM mannitol in ½MS medium to simulate drought-induced osmotic stress, and RNA-seq libraries were prepared from samples collected at 0, 2, 6, 12, and 24 h post-treatment; Figure S5. Validation and expression analysis of *StCML50* transgenic Arabidopsis. (A) Positive screening of transgenic seeds on ½MS medium supplemented with 50 μM hygromycin. Seedlings with well-developed true leaves and normal root growth were designated as positive transgenic plants. (B) PCR verification of transgenic plants. Lane M: DL2000 DNA marker; Lanes 1–15: Independent transgenic lines; Lane WT: Wild-type (WT) plants; Lane P: pCAMBIA1304-StCML50 plasmid (positive control). Lane CK: Nuclease-free water (negative control); (C) Expression analysis of *StCML50* in transgenic lines and WT plants. The Arabidopsis Actin gene served as the internal reference gene. Data are presented as the mean ± standard deviation (SD) (*n* = 3 independent biological replicates). ** indicates a significant difference at the *p* < 0.01 level; Table S1: Basic molecular characteristics of the potato *StCML* gene family. Table S2: The conserved motifs of the *CML* gene family; Table S3: Syntenic gene pairs of the potato *StCML* gene family with other species; Table S4: The sequences of primers were used for *StCMLs* in qRT-PCR.
