# Supplementary material for: Heterologous Expression of the StCML50 Gene Enhances Drought Tolerance in Transgenic Arabidopsis
Source: Plants (Basel). 2026 Jan 29;15(3):417. doi: 10.3390/plants15030417 (PMC12899910; doi:10.3390/plants15030417)
Supplement: Supplementary file 1 [file plants-15-00417-s001.zip › Supplementary figures.pdf]

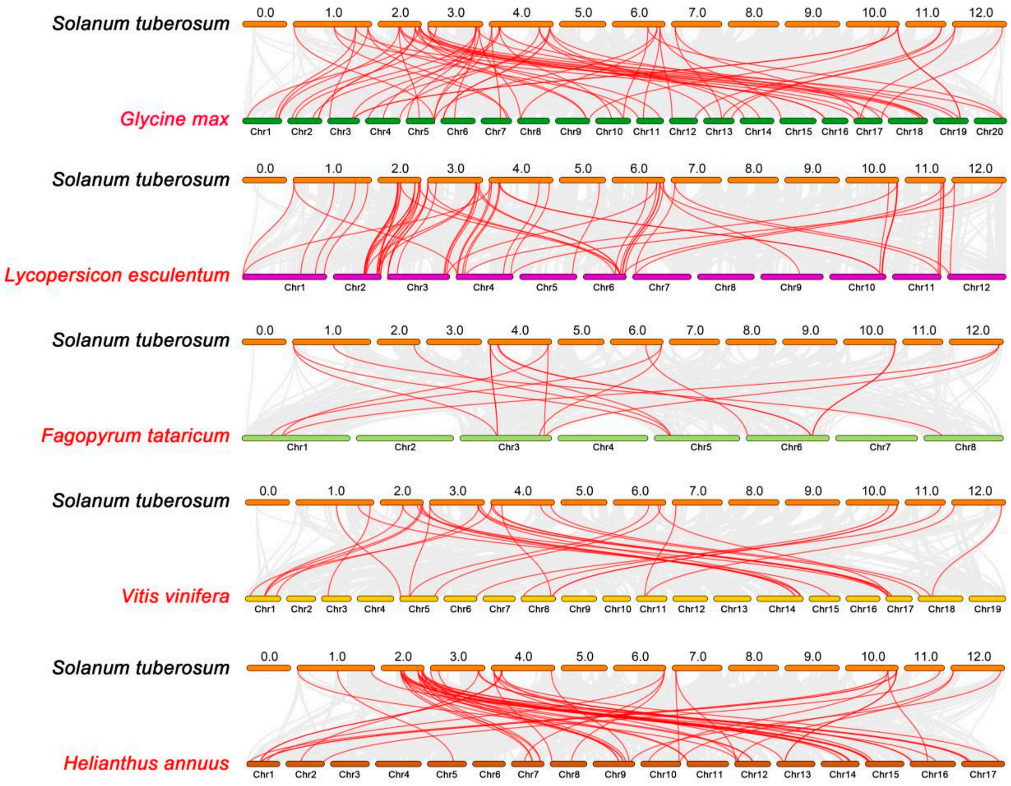

Figure S1. Synteny relationships of *CML* genes between potato and five other plants species. Gray lines in the background represent genome-wide collinear blocks across the genomes of the six species, while red lines specifically highlight syntenic *CML* gene pairs between potato and the other five plants.

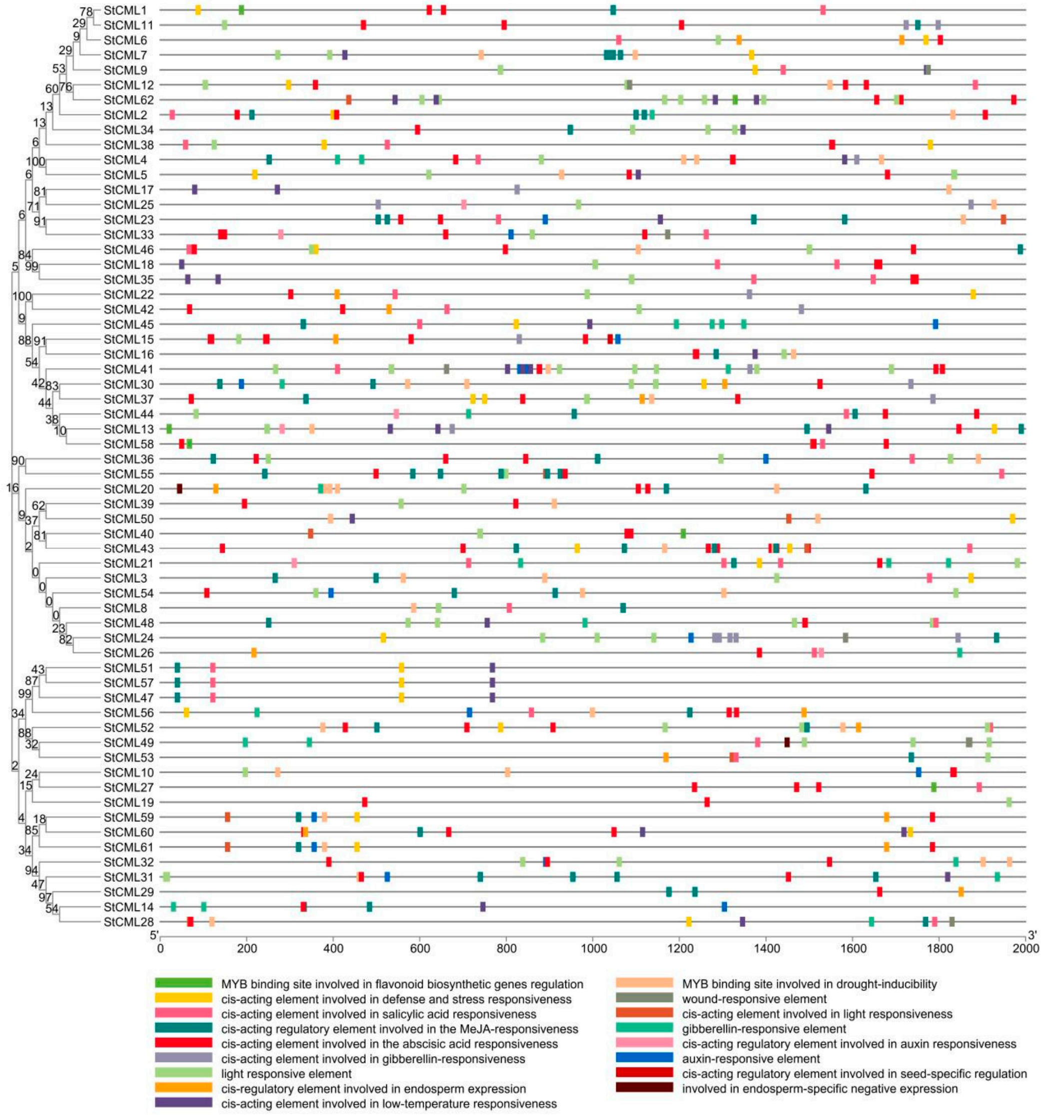

Figure S2. Analysis of *cis*-regulatory elements in the *StCML* gene family. The distribution of predicted *cis*-regulatory elements within the 2.0-kb upstream promoter regions of *StCML* family genes was analyzed, with a specific focus on elements associated with environmental stress responses.

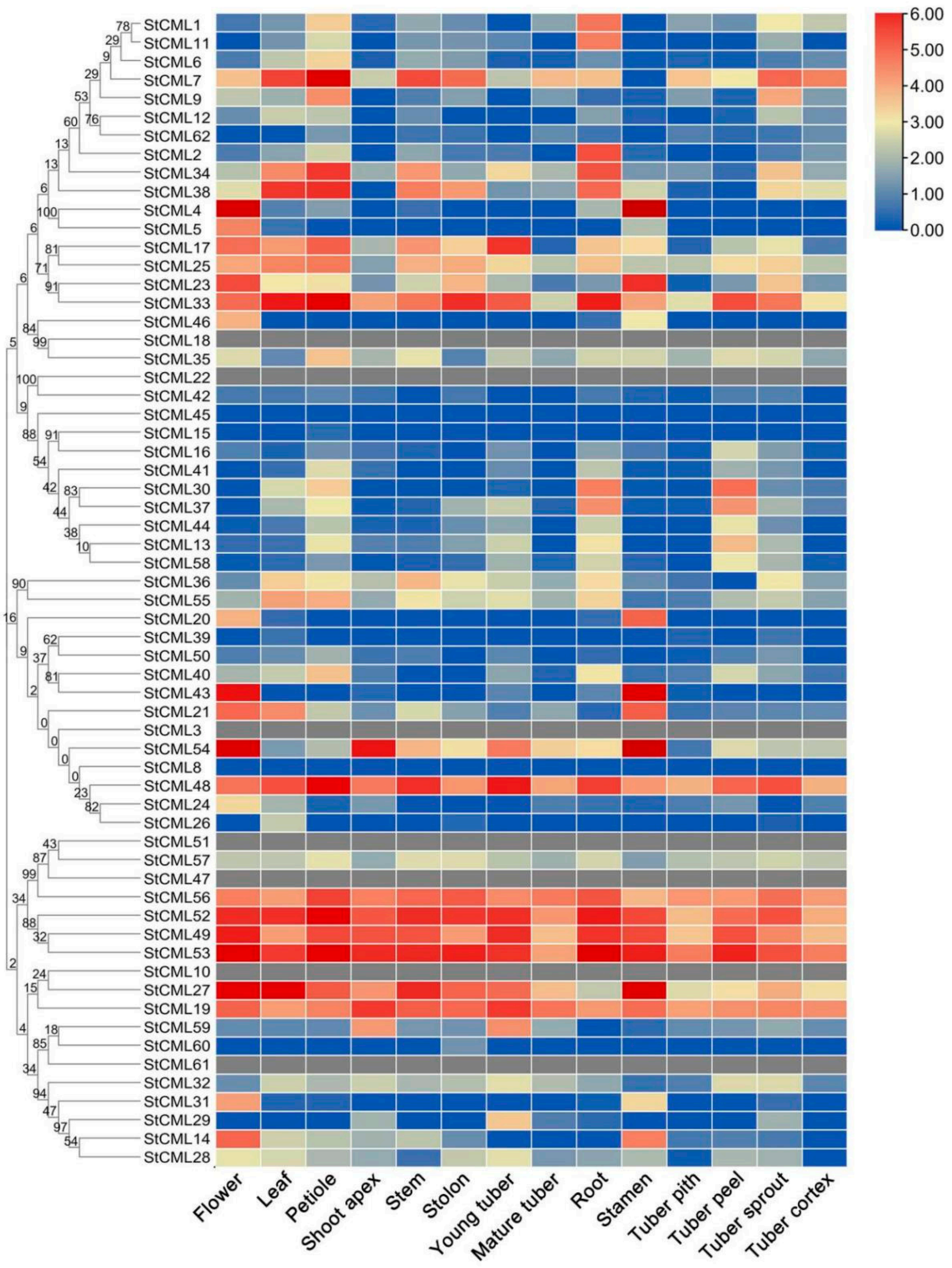

Figure S3. Tissue-specific expression profiling of *StCML* gene family members. Hierarchical clustering analysis was performed on the transcript abundance of *StCML* genes across multiple potato tissues, and results are presented as a heatmap. Expression levels were normalized via $\log_2$(FPKM + 1), where FPKM denotes fragments per kilobase of transcript per million mapped reads. The color gradient represents relative expression intensities, with a color scale bar denoting relative fold-change values.

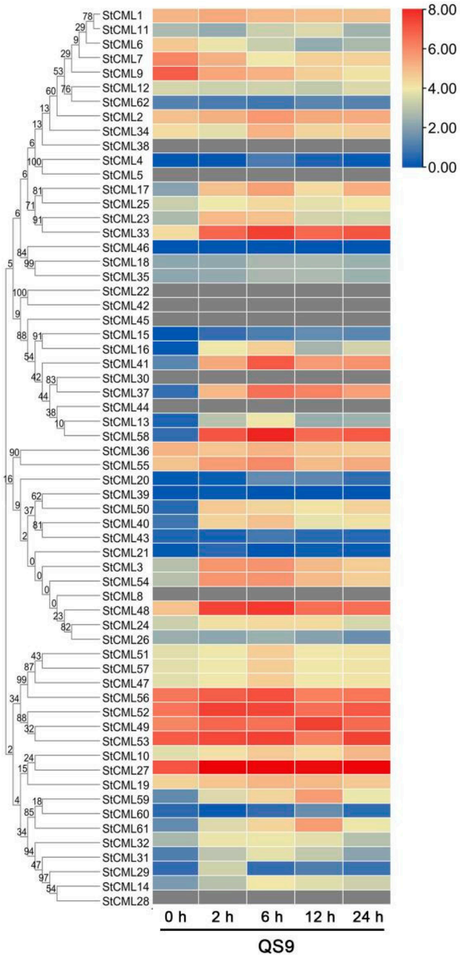

Figure S4. Transcriptomic analysis of *StCML* genes in potato under drought stress. Expression profiles (normalized as $\log_2$[FPKM+1]) were generated for the drought-tolerant potato cultivar ‘Qingshu NO.9’ (QS9). Three-week-old in vitro-grown potato seedlings were treated with 200 mM mannitol in ½MS medium to simulate drought-induced osmotic stress, and RNA-seq libraries were prepared from samples collected at 0, 2, 6, 12, and 24 h post-treatment.

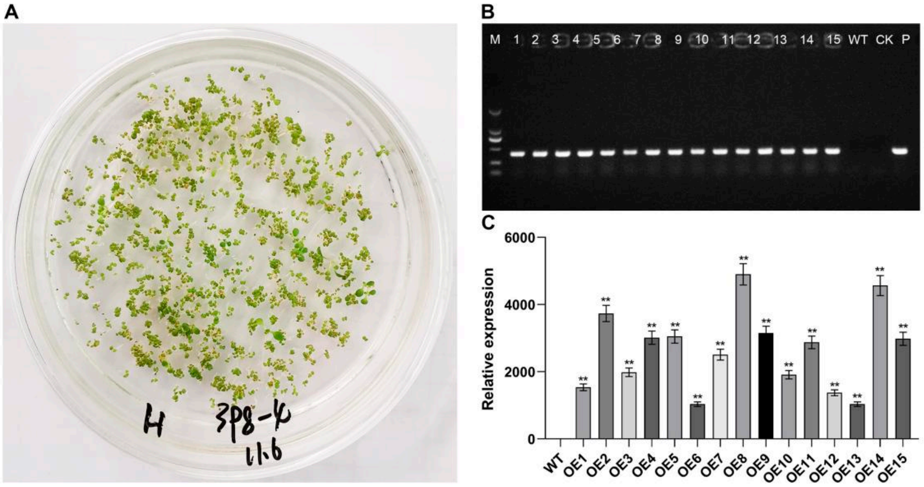

Figure S5. Validation and expression analysis of *StCML50* transgenic Arabidopsis. (A) Positive screening of transgenic seeds on ½MS medium supplemented with 50 μM hygromycin. Seedlings with well-developed true leaves and normal root growth were designated as positive transgenic plants. (B) PCR verification of transgenic plants. Lane M: DL2000 DNA marker; Lanes 1-15: Independent transgenic lines; Lane WT: Wild-type (WT) plants; Lane P: pCAMBIA1304-StCML50 plasmid (positive control). Lane CK: Nuclease-free water (negative control); (C) Expression analysis of *StCML50* in transgenic lines and WT plants. The Arabidopsis *Actin* gene served as the internal reference gene. Data are presented as the mean ± standard deviation (SD) (n = 3 independent biological replicates).. ** indicates a significant difference at the $P <$ 0.01 level.
