# Supplementary material for: Heterologous Expression of the StCML50 Gene Enhances Drought Tolerance in Transgenic Arabidopsis
Source: Plants (Basel). 2026 Jan 29;15(3):417. doi: 10.3390/plants15030417 (PMC12899910; doi:10.3390/plants15030417)
Supplement: Supplementary file 1 [file plants-15-00417-s001.zip › Supplementary table S4.pdf]

Table S4. The sequences of primers were used for *StCMLs* in qRT-PCR.

Gene	Sequences
AtRD29A	F: 5'- TCGCCACATTCTGTTGAAGAGGCT -3'
	R: 5'- TGGAGCCAAGTGATTGTGGAGACT -3'
AtRD29B	F: 5'- GGCATTTGGTCTAAGGTGAGAA -3'
	R: 5'- CAGACT CGATCCGCTGGTA -3′
AtRD22	F: 5'- TTCTCTTCCTA GTCAAAGGCTTT -3'
	R: 5'- GCAGACTTTGGC ACCGTGCT -3'
AtDREB2	F: 5'- GACGGCGACGGTATGGTTCAC -3'
	R: 5'- GGGAAAACAGGGCGACCCAAAG -3'
AtCOR15A	F: 5'- GTGCGGTTTGCTAGTTCCCTTT -3'
	R: 5'- ACCCTACAGCCCAAAAGCTACA -3'
AtKIN	F: 5'- GATGATAAGCTACTCCAGACCC -3'
	R: 5'- TTGTTGTGGTGAGCACATTTAG -3'
AtPP2C2	F: 5'- AGGAAACATCACTGTTGGAGAT -3'
	R: 5'- GAGTTTGGTCCAGTAAGAGGAA -3'
AtP5CS	F: 5'-CGAAAAGGACTCAACACAAGAG-3'
	R: 5'- CGAGTTATCGTAAAGCCTACCT-3'
AtActin	F: 5'- CCACATGCTATTCTGCGTTTGGACC -3'
	R: 5'- CATCCCTTACGATTTCACGCTCTGC -3'
StActin	F: 5'- AGGAGCATCCTGTCCTCCTAA -3'
	R: 5'- CACCATCACCAGAGTCCAACA -3'
